# Acute exacerbation of rheumatoid arthritis-associated interstitial lung disease: mortality and its prediction model

**DOI:** 10.1186/s12931-022-01978-y

**Published:** 2022-03-11

**Authors:** Hironao Hozumi, Masato Kono, Hirotsugu Hasegawa, Shinpei Kato, Yusuke Inoue, Yuzo Suzuki, Masato Karayama, Kazuki Furuhashi, Noriyuki Enomoto, Tomoyuki Fujisawa, Naoki Inui, Yutaro Nakamura, Koshi Yokomura, Hidenori Nakamura, Takafumi Suda

**Affiliations:** 1 Second Division, Department of Internal Medicine, Hamamatsu University School of Medicine, 1-20-1 Handayama Higashiku, Hamamatsu, 431-3192 Japan; 2 Department of Respiratory Medicine, Seirei Hamamatsu General Hospital, 2-12-12 Sumiyoshi, Naka Ward, Hamamatsu, 430-8558 Japan; 3 Department of Respiratory Medicine, Seirei Mikatahara General Hospital, 3453 Mikatahara-cho, Kita-ku, Hamamatsu, 433-8558 Japan; 4 Department of Clinical Pharmacology and Therapeutics, Hamamatsu University School of Medicine, 1-20-1 Handayama Higashiku, Hamamatsu, 431-3192 Japan

**Keywords:** Rheumatoid arthritis, Interstitial lung disease, Acute exacerbation, Mortality, Prognostic factor, Mortality prediction model, Prognostic classification

## Abstract

**Background:**

Patients with rheumatoid arthritis-associated interstitial lung disease (RA-ILD), like those with idiopathic pulmonary fibrosis (IPF), might develop an unexpected acute exacerbation (AE)—a rapidly progressing and deadly respiratory decline. Although AE incidence and risk factors in RA-ILD patients are known, their post-AE clinical course remains unknown owing to the rarity of AE-RA-ILD. This multicentre retrospective study evaluated post-AE mortality and prognostic variables in AE-RA-ILD patients and created a mortality prediction model for AE-RA-ILD.

**Methods:**

This research comprised 58 patients with AE-RA-ILD and 96 with AE-IPF (a control disease). Multivariate Cox regression analysis was performed to identify prognostic variables. A prediction model was created with recursive partitioning (decision tree).

**Results:**

The post-AE 90-day mortality rate in the overall AE-RA-ILD group was 48.3%; percent predicted forced vital capacity within 12 months before AE onset (baseline %FVC) and PaO_2_/FiO_2_ ratio at AE onset (P/F at AE) were independent predictors of mortality. Post-AE 90-day mortality rates were 40.6% and 43.8%, respectively, in AE-RA-ILD and AE-IPF patients propensity score-matched for age, sex, baseline %FVC and P/F at AE (*P* = 1.0000). In AE-RA-ILD patients, *C*-indices of baseline %FVC and P/F at AE to predict post-AE 90-day mortality were 0.604 and 0.623, respectively. A decision tree model based on these prognostic factors classified AE-RA-ILD patients into mild, moderate and severe groups (post-AE 90-day mortality rates: 20.8%, 64.0% and 88.9%, respectively; *P* = 0.0002); the *C*-index improved to 0.775.

**Conclusions:**

Post-AE mortality was high in AE-RA-ILD patients similar to AE-IPF patients. The discovered prognostic factors and our mortality prediction model may aid in the management of AE-RA-ILD patients.

**Supplementary Information:**

The online version contains supplementary material available at 10.1186/s12931-022-01978-y.

## Background

Rheumatoid arthritis (RA) is an inflammatory autoimmune disease that predominantly affects the joints [1, 2]. Interstitial lung disease (ILD) is a frequent extra-articular manifestation in RA patients and is linked to morbidity and mortality [3]. According to recent research, although overall RA-related mortality has reduced, RA-ILD mortality has not, indicating that enhanced RA care may have resulted in better overall outcomes but has limited therapeutic effect for the RA-ILD subgroup [4]. Hence, advances in the understanding and management of RA-ILD are required.

Acute exacerbation (AE) of idiopathic pulmonary fibrosis (IPF) is a rapidly progressing, deadly respiratory decline defined by new extensive alveolar abnormalities superimposed on underlying pulmonary fibrosis [5, 6]. AE develops unexpectedly in IPF patients over time, with a high post-AE 90-day mortality rate of around 30–60% [5]. Lower baseline lung function [e.g. % predicted forced vital capacity (%FVC)] before AE onset and lower PaO_2_/FiO_2_ ratio at AE onset (P/F at AE) are linked to an increased risk of mortality in AE-IPF patients [5, 7–10]. AE can also develop in patients with ILDs other than IPF, such as connective tissue disease (CTD)-related ILD [7, 11–14]. One of the most common CTD-ILDs linked with AE is RA-ILD [15]. The incidence of AE and its risk factors in RA-ILD patients have been documented [13, 16]; however, there is little data on post-AE mortality and prognostic variables in those who developed AE (AE-RA-ILD) because data are mostly based on case series/small studies. Furthermore, no mortality prediction model for AE-RA-ILD has been developed.

The aims of this study were to compare the post-AE mortality and cumulative survival rates of AE-RA-ILD patients with those of AE-IPF patients, a control illness, and to clarify prognostic factors for AE-RA-ILD and establish a mortality prediction model based on these factors.

## Methods

### Patients and diagnostic criteria

This multicentre retrospective study enrolled consecutive RA-ILD and IPF patients who were diagnosed with a first episode of AE between 2007 and 2019 at the Hamamatsu University, Seirei Mikatahara General or Seirei Hamamatsu General Hospitals. RA was diagnosed by rheumatologists based on the 1987 American College of Rheumatology (ACR) classification criteria or the 2010 ACR/European League Against Rheumatism classification (EULAR) criteria [1, 2]. Fibrosing ILD (i.e. ILD in this study) was defined as the presence of bilateral reticular opacities with/without traction bronchiectasis on chest high-resolution computed tomography (HRCT) and was diagnosed based on the consensus between radiologists and pulmonologists. The diagnosis of IPF was confirmed through retrospective multidisciplinary discussion based on the 2018 IPF guideline [6]. The diagnosis of AE was reassessed for this study. AE-IPF was diagnosed based on the 2016 AE-IPF International Working Group report criteria [5]. As in recent studies on AE-ILD other than AE-IPF [7, 14, 17–19], AE-RA-ILD was diagnosed based on the 2016 AE-IPF criteria with slight modifications, as events meeting all the following criteria: (1) the presence of fibrosing ILD on previous HRCT; (2) acute worsening or development of dyspnoea typically within 1 month; (3) new bilateral ground-glass opacity and/or consolidation superimposed on a background pattern consistent with fibrosing ILD on HRCT and 4) deterioration that is not fully explained by cardiac failure or fluid overload [5] (Additional file 1: Fig. S1). The 2016 AE-IPF criteria no longer require the exclusion of triggers such as infection or ‘drug toxicity’, except for cardiac failure/fluid overload. Therefore, we diagnosed AE if the patients fulfilled the criteria, even if they were undergoing treatment with any drug, including methotrexate (MTX). The patient inclusion criteria were as follows: both HRCT findings and %FVC within 12 months before AE onset (baseline HRCT and baseline %FVC, respectively) were available. Consequently, 58 AE-RA-ILD patients and 96 AE-IPF patients were enrolled in this study. Baseline HRCT patterns were classified based on the 2018 IPF guideline as usual interstitial pneumonia (UIP) (Additional file 1: Fig. S2), probable UIP, indeterminate for UIP and alternative diagnosis patterns [6]. This multicentre study was conducted in accordance with the Declaration of Helsinki and was approved by the institutional review board of each participating institution (Hamamatsu University School of Medicine [approval number: 19-206], Seirei Mikatahara General Hospital [approval number: 19-42] and Seirei Hamamatsu General Hospital [approval number: 3211]). Written informed consent was not required owing to the retrospective nature of the study.

### Data collection

Data pertaining to the following variables were collected from the medical records: clinical data; results of pulmonary function tests, including baseline %FVC and predicted diffusing capacity of the lung carbon monoxide within 12 months before AE onset (baseline %DL_CO_); laboratory data at AE onset, including serum C-reactive protein, Krebs von den Lungen-6 (KL-6) and P/F; HRCT; treatment details and outcomes. The observation period lasted from the date of AE onset until the last visit (the date of censoring/mortality). Patients were censored if they remained alive until 31 March 2021.

### Statistical analyses

All data were analysed using JMP version 13.2.1 (SAS Institute Inc., NC, USA), R software version 4.0.2 (The R Foundation for Statistical Computing, Austria) and Prism version 7.04 (GraphPad Software Inc., CA, USA). *P* < 0.05 was considered to indicate statistical significance. List-wise deletion was performed when handling missing data. Continuous and categorical variables were expressed as median [interquartile range (IQR)] and number (%), respectively. The Wilcoxon/Kruskal–Wallis test and Fisher’s exact test or Chi-squared test were performed for between-group comparisons. The cumulative survival rates were calculated using the Kaplan–Meier test, and the Wilcoxon test was used to assess the between-group differences. Cox proportional hazards regression analysis was used to identify the prognostic factors. Thereafter, hazard ratio (HR), 95% confidence interval (CI) and* P* values were calculated. Age, sex, baseline %FVC, P/F at AE and all variables that showed a significant association in the univariate analysis were included in the multivariate model. Variables that did not show any significant association in the univariate analysis were re-evaluated after adjustment. Propensity score matching was performed to compare patients with AE-RA-ILD and those with AE-IPF. Briefly, propensity scores, which predict the probabilities of each patient being assigned to AE-RA-ILD or AE-IPF, were calculated using a logistic regression model adjusted for age, sex and identified prognostic factors. Matching was performed using the following algorithm: 1:1 nearest neighbour matching with a ± 0.05 calliper and no replacement. Receiver-operating characteristic (ROC) analysis was performed to determine an optimal cut-off value of continuous variables (Youden index). A mortality prediction model was generated on the basis of recursive partitioning creating a decision tree. Variables such as age, sex and identified prognostic factors were determined as candidate variables for splitting, and node splitting was based on the LogWorth statistics [− log_10_ (*P*-value)], whereas the candidate variable for the split that maximises LogWorth was determined to be the optimal split, as reported in candidate reports. The splitting process was terminated when the study cohort was divided into three groups: mild, moderate and severe. The discrimination performance of the models was evaluated using the concordance statistic (*C*-index).

## Results

### Characteristics

Table 1 presents the patient characteristics. AE-RA-ILD patients did not differ significantly from AE-IPF patients in terms of age and P/F at AE, but they had a greater proportion of women, a higher baseline %FVC and a lower number of patients with UIP pattern on HRCT. All patients in both the AE-RA-ILD and AE-IPF groups received corticosteroids as first-line therapy for AE; however, the proportion of AE-RA-ILD patients who were simultaneously treated with an immunosuppressant was greater in the AE-RA-ILD group than in the AE-IPF group.

**Table 1:** Characteristics of AE-RA-ILD and AE-IPF patients

	AE-RA-ILD N = 58	AE-IPF N = 96	*P*-value
Age, years^a^	73 (68–80)	74 (69–79)	0.8244
Sex, male	35 (60.3)	81 (84.4)	0.0011
Smoking, ever^a^	39 (67.2)	80 (83.3)	0.0286
Baseline %FVC^b^	81.7 (66.2–90.6)	69 (54.2–84.4)	0.0059
Baseline %DL_CO_^b, c^	59.9 (47.1–75.4)	58.5 (43.0–78.5)	0.9939
P/F at AE, Torr^a^	220 (163–285)	223 (163–276)	0.7883
C-reactive protein, mg/dL^a^	9.4 (5.7–14.9)	11.4 (8.3–17.2)	0.3654
Rheumatoid factor, U/mL^a^	116 (37–322)	–	–
ACPA, positive^b, d^	41 (89.1)	–	–
KL-6, U/mL^a^	1042 (739–1559)	1530 (973–2456)	0.0013
Baseline HRCT pattern^b^			< 0.0001
UIP	34 (58.6)	85 (88.5)	
Probable UIP	10 (17.2)	11 (11.5)	
Indeterminate for UIP	3 (5.2)	0 (0)	
Alternative	11 (19.0)	0 (0)	
Infection-triggered AE^e^	5 (8.6)	12 (12.5)	0.5983
Treatment for AE			
Methylprednisolone pulse	58 (100)	96 (100)	1.0000
Prednisolone	58 (100)	96 (100)	1.0000
Immunosuppressant	31 (53.5)	34 (35.4)	0.0304
Intravenous cyclophosphamide	19 (32.8)	32 (33.3)	1.0000
Calcineurin inhibitor	13 (22.4)	2 (2.1)	< 0.0001
Post-AE observation period, day	121 (22–755)	236 (28–477)	0.8741
Mortality within 90 days after AE onset	28 (48.3)	34 (35.4)	0.1292
Mortality during study period	43 (74.1)	82 (85.4)	0.0925
Respiratory condition-related mortality	37 (63.8)	71 (74.0)	0.2058

Data presented as median (interquartile range) or as number (%)

*AE* acute exacerbation, *RA* rheumatoid arthritis, *ILD* interstitial lung disease, *IPF* idiopathic pulmonary fibrosis, *HRCT* high-resolution computed tomography, *UIP* usual interstitial pneumonia, *%FVC* percent predicted forced vital capacity, *%DL*_*CO*_ percent predicted diffusing capacity of the lung for carbon monoxide, *P/F* PaO_2_/FiO_2_ ratio, *ACPA* anti-cyclic citrullinated peptide antibody, *KL-6* Krebs von den Lungen-6

^a^At AE onset

^b^Within 12 months before AE

^c^RA, n = 34; IPF, n = 62

^d^n = 46

^e^Due to an apparent infection with an identifiable cause that occurred within 1 month before AE onset

**Fig. 1:**
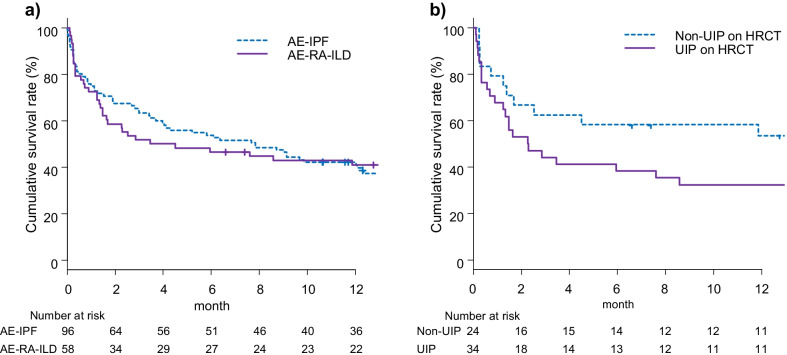
Post-AE survival curves. **a** Post-AE cumulative survival rates at 3, 6 and 12 months were 63.2%, 53.7% and 42.1% in patients with AE-IPF, respectively, and 51.7%, 46.6% and 41.0% in patients with AE-RA-ILD, respectively (*P* = 0.9090). **b** Post-AE cumulative survival rates at 3, 6 and 12 months were 62.5%, 58.3% and 53.5% in AE-RA-ILD patients with the non-UIP pattern on HRCT, respectively, and 44.1%, 38.2% and 32.4% in AE-RA-ILD patients with UIP pattern on HRCT, respectively (*P* = 0.1747). *AE* acute exacerbation, *IPF* idiopathic pulmonary fibrosis, *RA* rheumatoid arthritis, *ILD* interstitial lung disease, *UIP* usual interstitial pneumonia, *HRCT* high-resolution computed tomography

Of the 58 AE-RA-ILD patients, 28 died within 90 days of disease onset [i.e. post-AE 90-day mortality rate 48.3% (95% CI 35.9–60.8%)] and 43 (74.1%) died during the post-AE observation period. The post-AE cumulative survival rates at 3, 6 and 12 months were 51.7%, 46.6% and 41.0%, respectively, in the overall AE-RA-ILD group, versus 63.2%, 53.7% and 42.1%, respectively, in the overall AE-IPF group; there was no significant between-group difference (*P* = 0.9090; Fig. 1A). In the AE-RA-ILD group, the leading cause of mortality was respiratory conditions, including first AE (28 patients), AE relapse (6 patients), chronic respiratory failure after AE (3 patients), infection (5 patients) and unknown cause (1 patient); respiratory condition-related mortality accounted for a higher proportion of all-cause mortality, as it did in AE-IPF patients. The post-AE 90-day mortality rates of AE-RA-ILD patients with UIP patterns on HRCT at baseline and those with non-UIP patterns on HRCT were 55.9% and 41.7%, respectively (*P* = 0.4242). The post-AE cumulative survival rate in AE-RA-ILD patients with UIP patterns on HRCT was lower than in those with non-UIP patterns on HRCT, although the difference was not statistically significant (*P* = 0.1747) (Fig. 1B).

The Additional file 1: Table S1 shows the treatment for RA before the onset of AE in AE-RA-ILD patients. Of the 58 patients, 52 (89.7%) had been treated for RA prior to AE onset, and those therapies had been continued for at least 6 months before AE onset. Thirty-three patients (56.9%) received 7.5 mg/day of oral prednisolone (PSL); however, no patient received > 7.5 mg/day. Twenty-one patients (36.2%) received MTX, seven (12.1%) received tacrolimus, four (6.9%) received mizoribine, one (1.7%) received tofacitinib, eight (13.8%) received bucillamine, thirteen (22.4%) received salazosulfapyridine and three (5.2%) received iguratimod. Six patients (10.3%) were treated with biological therapy, including abatacept (2 patients), etanercept (2 patients) and tocilizumab (2 patients).

### Prognostic factors of AE-RA-ILD

Table 2 shows the findings of the Cox proportional hazards analysis of all-cause mortality. Lower baseline %FVC and lower P/F at AE were shown to be independent prognostic variables in the multivariate analysis. Table 3 shows the findings of multivariate models adjusted for age, sex, baseline %FVC and P/F at AE. UIP patterns on HRCT (vs. non-UIP patterns on HRCT) tended to be associated with increased mortality. Treatment for RA before the onset of AE, including MTX, were not related to mortality. The concurrent immunosuppressant and corticosteroid use for AE did not have a significant effect on mortality in AE-RA-ILD patients (Tables 2 and 3), which was similar to that in AE-IPF (a control disease) patients (Additional file 1: Table S2).

**Table 2:** Cox proportional hazards regression analysis of all-cause mortality

Variables	Univariate model			Multivariate model		
	HR	95% CI	*P*-value	HR	95% CI	*P*-value
Age, per 1 year increase^a^	1.02	0.99–1.06	0.2330	1.02	0.97–1.06	0.4675
Sex, male (vs. female)	0.66	0.36–1.22	0.1845	0.41	0.16–1.05	0.0622
Smoking, ever (vs. never)^a^	0.63	0.34–1.20	0.1539			
Baseline %FVC, per 1% increase^b^	0.97	0.95–0.99	0.0202	0.97	0.95–0.99	0.0168
Baseline %DL_CO_, per 1% increase^b, c^	0.99	0.97–1.01	0.3608			
P/F at AE, per 10 Torr increase^a^	0.96	0.92–0.99	0.0407	0.94	0.88–0.99	0.0240
C-reactive protein, per 1 mg/dL^a^	1.03	0.99–1.06	0.0912			
Rheumatoid factor, per 10 U/mL increase^a^	1.00	0.99–1.01	0.6296			
ACPA, positive (vs. negative)^b, d^	1.11	0.40–4.65	0.8572			
KL-6, per 100 U/mL increase^a^	0.97	0.93–1.002	0.0720			
UIP pattern on HRCT (vs. other patterns)^b^	1.45	0.79–2.76	0.2309			
Infection-triggered AE^e^	1.98	0.58–5.14	0.2453			
Treatment for RA before AE, yes (vs. no)						
Prednisolone ≤ 7.5 mg/day	3.33	0.75–14.8	0.1086			
Methotrexate	0.73	0.37–1.37	0.3366			
Tacrolimus	1.24	0.47–2.75	0.6340			
Bucillamine	2.08	0.84–4.45	0.1078			
Salazosulfapyridine	1.32	0.63–2.55	0.4416			
Treatment for AE (vs. CS monotherapy)						
CS + IS	1.05	0.57–1.92	0.8826			
CS + IVCY	1.36	0.68–2.70	0.3758			
CS + CNI	0.62	0.26–1.35	0.2349			

*%FVC* percent predicted forced vital capacity, *%DL*_*CO*_ percent predicted diffusing capacity of the lung for carbon monoxide, *P/F* PaO_2_/FiO_2_ ratio, *ACPA* anti-cyclic citrullinated peptide antibody, *KL-6* Krebs von den Lungen-6, *UIP* usual interstitial pneumonia, *HRCT* high-resolution computed tomography, *RA* rheumatoid arthritis, *AE* acute exacerbation, *CS* corticosteroids, *IS* immunosuppressant, *IVCY* intravenous cyclophosphamide, *CNI* calcineurin inhibitor, *HR* hazard ratio, *CI* confidence interval

^a^At AE onset

^b^Within 12 months before AE

^c^n = 34

^d^n = 46

^e^Due to an apparent infection with an identifiable cause that occurred within 1 month before AE onset

**Table 3:** Adjusted multivariate model of all-cause mortality

	HR	95% CI	*P*-value
Smoking, ever (vs. never)^a^	0.97	0.25–3.63	0.9640
C-reactive protein, per 1 mg/dL^a^	0.99	0.93–1.04	0.5770
Rheumatoid factor, per 10 U/mL increase^a^	5.93	0.65–50.56	0.1110
ACPA, positive (vs. negative)^b, c^	0.29	0.07–1.53	0.1329
KL-6, per 100 U/mL increase^a^	0.99	0.94–1.02	0.4416
UIP pattern on HRCT (vs. other patterns)^b^	1.84	0.99–3.56	0.0548
Infection-triggered AE^d^	3.16	0.87–9.28	0.0760
Treatment for RA before AE, yes (vs. no)			
Prednisolone ≤ 7.5 mg/day	6.78	0.47–111.3	0.1595
Methotrexate	1.16	0.38–3.18	0.7806
Tacrolimus	0.83	0.27–2.28	0.7224
Bucillamine	1.81	0.64–4.51	0.2468
Salazosulfapyridine	0.69	0.23–1.78	0.4491
Treatment for AE (vs. CS monotherapy)			
CS + IS	1.13	0.47–2.81	0.7858
CS + IVCY	1.67	0.62–4.56	0.3108
CS + CNI	0.91	0.28–2.77	0.8741

Adjusted for age, sex, baseline %FVC, and PaO_2_/FiO_2_ ratio

*ACPA* anti-cyclic citrullinated peptide antibody, *KL-6* Krebs von den Lungen-6, *UIP* usual interstitial pneumonia, *HRCT* high-resolution computed tomography, *RA* rheumatoid arthritis, *AE* acute exacerbation, *CS* corticosteroids, *IS* immunosuppressant, *IVCY* intravenous cyclophosphamide, *CNI* calcineurin inhibitor, *HR* hazard ratio, *CI* confidence interval

^a^At AE onset

^b^Within 12 months before AE

^c^n = 46

^d^Due to an apparent infection with an identifiable cause that occurred within 1 month before AE onset

### Propensity score-matched AE-RA-ILD and AE-IPF

**Fig. 2:**
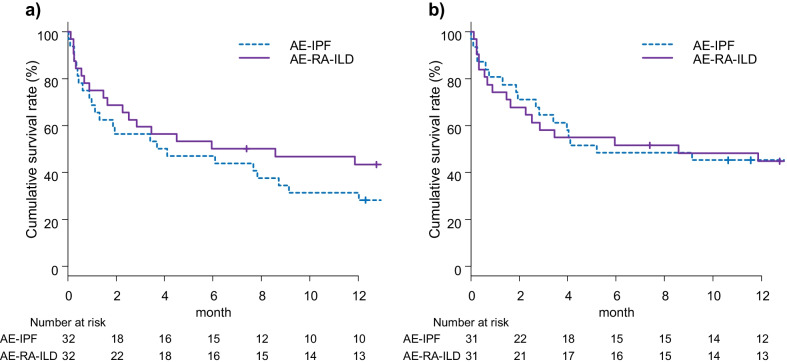
Post-AE survival curves of propensity score-matched AE-RA-ILD and AE-IPF. **a** After propensity score matching for age, sex, baseline %FVC and P/F at AE, post-AE cumulative survival rates at 3, 6 and 12 months were 56.2%, 46.9% and 31.5% in patients with AE-IPF, respectively, and 59.4%, 50.0% and 43.3% in patients with AE-RA-ILD, respectively (*P* = 0.3217). **b** After propensity score matching for age, sex, baseline %FVC, P/F ratio and baseline HRCT pattern, post-AE cumulative survival rates at 3, 6 and 12 months were 64.5%, 48.4% and 45.2% in patients with AE-IPF, respectively, and 58.1%, 51.6% and 44.7% in patients with AE-RA-ILD, respectively (*P* = 0.7845). *AE* acute exacerbation, *RA* rheumatoid arthritis, *ILD* interstitial lung disease, *IPF* idiopathic pulmonary fibrosis, *%FVC* percent predicted forced vital capacity, *P/F* PaO_2_/FiO_2_ ratio, *HRCT* high-resolution computed tomography

Additional file 1: Table S3 shows patient characteristics of AE-RA-ILD and AE-IPF patients who were propensity score-matched for age, sex, baseline %FVC and P/F at AE. There was no statistically significant difference in post-AE 90-day mortality rates between AE-RA-ILD and AE-IPF patients (40.6% vs. 43.8%, respectively, *P* = 1.0000). In addition, there was no significant difference in post-AE cumulative survival rates between the groups (*P* = 0.3217; Fig. 2A). In Additional file 1: Table S4, patient characteristics of AE-RA-ILD and AE-IPF patients who were propensity score-matched for baseline HRCT pattern, in addition to age, sex, baseline %FVC and P/F at AE, are provided as a sensitivity analysis. There was no statistically significant difference in post-AE 90-day mortality rates between AE-RA-ILD and AE-IPF patients (41.9% vs. 35.5%, respectively, *P* = 0.7946). In addition, there was no significant difference in post-AE cumulative survival rates between the groups (*P* = 0.7845; Fig. 2B).

### Mortality prediction model

**Fig. 3:**
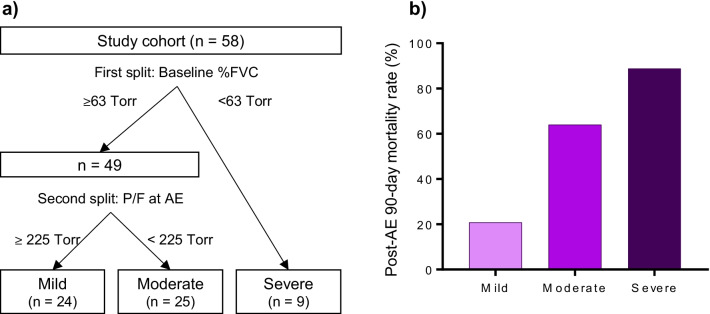
Mortality prediction model. **a** A decision tree predicting post-AE 90-day survival created by recursive partitioning. The splitting process was terminated when the study cohort was divided into the following three groups: mild, moderate and severe. The variables (cut-off points) of the optimal split in the first and second split candidates were baseline %FVC (63%) and P/F at AE (225 Torr), respectively. **b** The post-AE 90-day mortality rates of the patients classified by the decision tree: mild group, 20.8%, moderate group, 64.0% and severe group, 88.9% (*P* = 0.0002), with a discriminative performance (*C*-index) of 0.775. *%FVC* percent predicted forced vital capacity, *P/F* PaO_2_/FiO_2_ ratio, *AE* acute exacerbation

ROC analysis for predicting post-AE 90-day mortality revealed that the baseline %FVC and P/F at AE cut-off values were 63% and 225 Torr, respectively, with *C*-indices of 0.604 and 0.623. The post-AE 90-day mortality rate was higher in patients with baseline %FVC of < 63% than in those with baseline %FVC of ≥ 63% (88.9% vs. 42.9%, respectively; *P* = 0.0253) and in patients with P/F at AE of < 225 Torr than in those with P/F at AE of ≥ 225 Torr (67.7% vs. 29.6%, respectively; *P* = 0.0079). Figure 3A presents a decision tree constructed by recursive partitioning that predicts post-AE 90-day mortality. This model took into account age, sex, baseline %FVC, P/F at AE and baseline HRCT patterns. Additional file 1: Table S5 shows the candidate reports for the first and second splits. In the first and second splits, the variables (cut-off points) of the optimum split were baseline %FVC (63%) and P/F at AE (225 Torr), respectively. The post-AE 90-day mortality rates for the mild, moderate and severe groups were 20.8%, 64.0% and 88.9%, respectively (*P* = 0.0002; Fig. 3B), with a *C*-index of 0.775.

## Discussion

This is the most large-scale study yet to investigate post-AE mortality and prognostic variables for mortality, as well as the first to create a mortality prediction model for AE-RA-ILD. Furthermore, one of the study’s strengths is the use of physiological indicators linked with respiratory function, such as baseline %FVC and P/F at AE. The post-AE 90-day mortality rate in the overall AE-RA-ILD group was 48.3%. Lower baseline %FVC and lower P/F at AE were independent prognostic predictors in multivariate analysis. Post-AE 90-day mortality rates in both the AE-RA-ILD and AE-IPF groups were approximately 40% and comparable when propensity score-matched for age, sex, baseline %FVC and P/F at AE. The decision tree-based mortality prediction model, which used the identified prognostic factors, namely baseline %FVC and P/F at AE, was able to classify AE-RA-ILD patients into three groups with significantly different post-AE 90-day mortality rates: mild, moderate and severe, and its discriminative performance outperformed that of each prognostic factor alone.

Although studies have indicated that patients with AE-ILDs other than IPF, including CTD-ILD, have considerably lower post-AE mortality than patients with IPF [18, 20, 21], others have found that there was no meaningful prognostic difference [19, 22]. Because these studies grouped diverse ILD patients into one group and compared them with IPF patients without taking the severity of baseline ILD or AE into consideration, the results should be interpreted with caution. In addition, there has been little research on the post-AE clinical outcomes of RA-ILD patients. Park et al. reported that all three AE-RA-ILD patients who had a pathologic evidence of UIP died within 41 days after AE onset [11]. According to Song et al., 13 out of 14 AE-RA-ILD patients died, with a median duration from AE onset to mortality of 1.5 months [23]. Izuka et al. discovered in a single-centre investigation of 30 AE-RA-ILD patients that the post-AE 60-day mortality rate was approximately 40% [16]. However, the observation duration and diagnostic criteria for AE varied between studies, and the severity of baseline ILD or AE was not described. The present study, which was based on the AE criteria updated in 2016, compared AE-RA-ILD and AE-IPF patients using both crude and propensity score-matched comparisons and revealed that AE-RA-ILD patients have a post-AE 90-day mortality rate of > 40%, which is the same as that of IPF patients.

There are currently no prognostic predictors for post-AE mortality in AE-RA-ILD patients. Lower baseline %FVC, lower baseline %DL_CO_, lower P/F at AE and more severe CT abnormalities at AE have been linked to higher post-AE mortality in AE-IPF patients [5, 8–10]. These findings imply that both baseline lung function (e.g. degree of lung fibrosis) and acute oxygen status should be evaluated to better predict post-AE mortality [5, 7]. Regarding AE-RA-ILD, Izuka et al. reported that in univariate analysis, UIP pattern on HRCT was associated with a poor outcome, whereas current MTX use was associated with a better prognosis [16]; however, the study did not include background pulmonary function or oxygen status at the time of AE into the analysis and the sample size was small. Hence, the data must be validated by multivariate analysis in a larger cohort. In the present study, multivariate analysis revealed that UIP pattern on HRCT tended to be associated with a worse outcome in AE-RA-ILD patients, whereas MTX usage was not. Importantly, baseline %FVC and P/F at AE were independent predictive variables, which is consistent with previous studies on AE-IPF. It is proposed that such physiological parameters related to respiratory state might be universal prognostic variables regardless of AE-ILD background diseases. Further research on the prognostic relevance of these variables in other AE-ILDs might provide intriguing results.

There is no known therapy for AE-RA-ILD. High-dose corticosteroid therapy and supportive care, including high-flow oxygenation and invasive/non-invasive ventilation, are often used in clinical practice for such patients [7], with reference to the treatment of AE-IPF [5]. In recent years, the potential benefit of multimodal therapy for AE-IPF has been documented, including anti-fibrotic drugs, polymyxin-B direct hemoperfusion and lung transplantation [5]. There have been reports of these therapies being used on AE-RA-ILD patients [13, 18, 20, 22, 24, 25]; however, physicians must carefully evaluate the possible benefits against the risks of adverse events and higher medical expenses, as well as carefully determine the justifications for such treatments. In addition, certain patients may require end-of-life care [26]. In such cases, our mortality prediction model may be able to assist clinicians and patients/families in decision-making for treatment. Furthermore, clinical trials on acute respiratory distress syndrome have been conducted based on a severity classification model helpful for predicting mortality, which has considerably aided in the development of treatment options [27]. Our approach, we hope, will also be beneficial in the design of clinical trials to determine treatment options for AE-RA-ILD.

The notion of AE prevention is also therapeutically important to minimise AE-related mortality [5]. Disease-modifying anti-rheumatic drugs (DMARDs), such as corticosteroids, conventional DMARDs (e.g. MTX), biologic drugs and targeted synthetic DMARDs, may increase the risk of infection and drug-induced lung injury [3]. Joint surgery can result in ventilator-induced lung injury if performed under general anaesthesia and with a ventilator. Lung injury caused by infections, medications or surgery with positive-pressure mechanical ventilation can also cause AE [5]. Therefore, it is imperative to establish preventive management for AE induced by such RA-specific triggers. It has been observed that the likelihood of developing AE in patients with IPF and other fibrosing ILD is higher in those with a more physiologically and functionally advanced disease [5, 19]. In addition, patients with lower baseline %FVC had greater post-AE mortality, according to the present study. Hence, early treatment intervention to avoid the worsening of baseline pulmonary function may also be required. The INBUILD study, which included a range of progressive fibrosing ILDs, including RA-ILD, found that nintedanib, an anti-fibrotic drug, decreased pulmonary function loss [17]. Surprisingly, the study also indicated that nintedanib might lower the likelihood of developing AE. Another anti-fibrotic drug, pirfenidone, is being studied in a clinical trial for RA-ILD [28]. Given the significant mortality rate of AE-RA-ILD reported in this study, future research should focus not only on AE-RA-ILD therapy but also on creating RA-ILD-specific management to avoid AE and AE-related mortality.

The present study had several limitations. First, the retrospective design makes it susceptible to a variety of biases (e.g. due to missing data in ACPA titer, erythrocyte sedimentation rate, and RA disease activity index, we were unable to fully evaluate the association between these results and post-AE mortality). Second, the baseline %FVC utilised was tested within 12 months before AE onset, thus the variation in measurement time may have influenced the results. However, AE development is unpredictable, and measuring %FVC at AE onset is difficult; hence, it may be relatively feasible to utilise %FVC obtained within 12 months before AE onset. Third, our approach is inapplicable in instances when the %FVC is not determined before AE onset. In such situations, mortality should be predicted solely based on P/F at AE. However, because the present study discovered that baseline %FVC is a major prognostic factor in AE-RA-ILD patients, we recommend that %FVC be measured regularly in RA-ILD patients. Fourth, with the goal to build simple and feasible models based on clinical/physiological factors, the prognostic significance of HRCT assessment was not examined. The quantitative assessment of abnormalities on HRCT requires evaluation by a chest radiologist and thus may not be immediately useful in clinical practice. Finally, there is a wide range of therapies available for RA patients. Such treatment differences may have influenced the results.

## Conclusions

This multicentre cohort study discovered that AE-RA-ILD patients, like AE-IPF patients, had a higher post-AE mortality rate. Lower %FVC at baseline and lower P/F at AE were independent predictors of mortality. The simple mortality prediction model based on these prognostic factors outperformed each prognostic factor individually in predicting post-AE 90-day mortality. The prediction model allowed for the classification of AE-RA-ILD patients into three groups with significantly different prognoses: mild, moderate and severe. These findings will help guide physicians to determine a therapeutic strategy, assist patient/family decision-making and aid in the planning of future AE-RA-ILD research.

## Supplementary Information

**Additional file 1: Table S1.** Treatment for RA before AE onset. **Table S2.** Cox proportional hazards regression analysis: association between treatment for AE and all-cause mortality in AE-IPF (a control disease). **Table S3.** Patients with AE-RA-ILD and those with AE-IPF propensity score-matched for age, sex, baseline %FVC, and P/F at AE. **Table S4.** Patients with AE-RA-ILD and those with AE-IPF propensity score-matched for age, sex, baseline %FVC, P/F at AE, and baseline HRCT pattern. **Table S5.** Candidate reports. **Figure S1.** Representative AE images in a patient with RA-ILD. **Figure S2.** Representative image of UIP pattern on HRCT.

## Data Availability

The data that support the findings of this study are available from the Hamamatsu University School of Medicine, Seirei Mikatahara General Hospital and Seirei Hamamatsu General Hospital but restrictions apply to the availability of these data, which were used under license for the current study, and so are not publicly available. Data are however available from the authors upon reasonable request and with permission of the Hamamatsu University School of Medicine, Seirei Mikatahara General Hospital and Seirei Hamamatsu General Hospital.

## Abbreviations

ACPA: Anti-cyclic citrullinated peptide antibody
ACR: American College of Rheumatology
AE: Acute exacerbation
CI: Confidence interval
CS: Corticosteroids
CTD: Connective tissue disease
DMARD: Disease-modifying anti-rheumatic drug
EULAR: European League Against Rheumatism
HR: Hazard ratio
HRCT: High-resolution computed tomography
ILD: Interstitial lung disease
IPF: Idiopathic pulmonary fibrosis
IQR: Interquartile range
IS: Immunosuppressant
KL-6: Krebs von den Lungen-6
MTX: Methotrexate
P/F: PaO_2_ to FiO_2_ ratio
%DL_CO_: Percent predicted diffusing capacity of the lung carbon monoxide
%FVC: Percent predicted forced vital capacity
PSL: Prednisolone
RA: Rheumatoid arthritis
ROC: Receiver-operating characteristic
UIP: Usual interstitial pneumonia
