# Supplementary material for: Acute exacerbation of rheumatoid arthritis-associated interstitial lung disease: mortality and its prediction model
Source: Respir Res. 2022 Mar 11;23:57. doi: 10.1186/s12931-022-01978-y (PMC8915517; doi:10.1186/s12931-022-01978-y)
Supplement: Supplementary file 1 — Additional file 1: Table S1. Treatment for RA before AE onset. Table S2. Cox proportional hazards regression analysis: association between treatment for AE and all-cause mortality in AE-IPF (a control disease). Table S3. Patients with AE-RA-ILD and those with AE-IPF propensity score-matched for age, sex, baseline %FVC, and P/F at AE. Table S4. Patients with AE-RA-ILD and those with AE-IPF propensity score-matched for age, sex, baseline %FVC, P/F at AE, and baseline HRCT pattern. Table S5. Candidate reports. Figure S1. Representative AE images in a patient with RA-ILD. Figure S2. Representative image of UIP pattern on HRCT. [file 12931_2022_1978_MOESM1_ESM.docx]

**Table S1. Treatment for RA before AE onset**

|  | **N = 58** |
| --- | --- |
| Treatment for RA before AE, yes | 52 (89.7) |
| Prednisolone > 7.5 mg/day | 0 (0) |
| Prednisolone ≤ 7.5 mg/day | 33 (56.9) |
| Methotrexate | 21 (36.2) |
| Tacrolimus | 7 (12.1) |
| Mizoribine | 4 (6.9) |
| Tofacitinib | 1 (1.7) |
| Bucillamine | 8 (13.8) |
| Salazosulfapyridine | 13 (22.4) |
| Iguratimod | 3 (5.2) |
| Biological agent | 6 (10.3) |
| Abatacept | 2 (3.4) |
| Etanercept | 2 (3.4) |
| Tocilizumab | 2 (3.4) |

Data are presented as number (%)

AE, acute exacerbation; RA, rheumatoid arthritis

**Table S2. Cox proportional hazards regression analysis: association between treatment for AE and all-cause mortality in AE-IPF (a control disease)**

|  | **Univariate model** | | | **Multivariate model ^a^** | | |
| --- | --- | --- | --- | --- | --- | --- |
| Treatment for AE (vs. CS monotherapy) | **HR** | **95% CI** | ***P*-value** | **HR** | **95% CI** | ***P*-value** |
| CS + IS | 1.17 | 0.74–1.81 | 0.4979 | 1.03 | 0.64–1.63 | 0.9079 |
| CS + IVCY | 1.13 | 0.71–1.77 | 0.5950 | 1.02 | 0.63–1.63 | 0.9309 |
| CS + CNI | 2.23 | 0.36–7.33 | 0.3272 | 1.22 | 0.18–4.83 | 0.8064 |

^a^ Adjusted for age, sex, baseline %FVC, and PaO_2_/FiO_2_ ratio

AE, acute exacerbation; IPF, idiopathic pulmonary fibrosis; CS, corticosteroids; IS, immunosuppressant; IVCY, intravenous cyclophosphamide; CNI, calcineurin inhibitor; HR, hazard ratio; CI, confidence interval

**Table S3. Patients with AE-RA-ILD and those with AE-IPF propensity score-matched for age, sex, baseline %FVC and P/F at AE**

|  | **AE-RA-ILD**  **N = 32** | **AE-IPF**  **N = 32** | ***P*-value** |
| --- | --- | --- | --- |
| Age, years ^a^ | 73 (69–80) | 72 (68–78) | 0.8351 |
| Sex, male | 27 (84.4) | 24 (75.0) | 0.5356 |
| Smoking, ever ^a^ | 27 (84.4) | 26 (81.3) | 1.0000 |
| Baseline %FVC ^b^ | 78 (60–87) | 75 (54–87) | 0.7119 |
| Baseline %DL_CO_ ^b, c^ | 59.9 (43.8–75.4) | 53.9 (42.0–69.5) | 0.5593 |
| P/F at AE, Torr ^a^ | 234 (171–277) | 179 (157–251) | 0.1728 |
| C-reactive protein, mg/dL ^a^ | 8.3 (4.2–14.2) | 11.1 (5.3–16.6) | 0.7837 |
| KL-6, U/mL ^a^ | 1090 (834–1596) | 1898 (1162–2604) | 0.0080 |
| Baseline HRCT pattern ^b^ |  |  | 0.0400 |
| UIP | 20 (62.5) | 27 (84.4) |  |
| Probable UIP | 7 (21.9) | 5 (15.6) |  |
| Indeterminate for UIP | 1 (3.1) | 0 (0) |  |
| Alternative | 4 (12.5) | 0 (0) |  |
| Treatment for AE |  |  |  |
| Methylprednisolone pulse | 32 (100) | 32 (100) | 1.0000 |
| Prednisolone | 32 (100) | 32 (100) | 1.0000 |
| Immunosuppressant | 17 (53.1) | 17 (53.1) | 1.0000 |
| Intravenous cyclophosphamide | 13 (40.6) | 15 (46.9) | 0.8013 |
| Calcineurin inhibitor | 5 (15.6) | 2 (6.3) | 0.4258 |
| Post-AE observation period, day | 203 (32–756) | 119 (21–397) | 0.4282 |
| Mortality within 90 days after AE onset | 13 (40.6) | 14 (43.8) | 1.0000 |
| Mortality during study period | 23 (71.9) | 31 (96.9) | 0.0127 |
| Respiratory condition-related mortality | 18 (56.3) | 27 (84.4) | 0.0272 |

Data are presented as median (interquartile range) or as number (%)

^a^ At AE onset, ^b^ within 12 months before AE, ^c^ AE-RA-ILD, n = 26; AE-IPF n = 17

AE, acute exacerbation; RA, rheumatoid arthritis; ILD, interstitial lung disease; IPF, idiopathic pulmonary fibrosis; %FVC, percent predicted forced vital capacity; P/F, PaO_2_/FiO_2_ ratio; %DL_CO_, percent predicted diffusing capacity of the lung for carbon monoxide; KL-6, Krebs von den Lungen-6; HRCT, high-resolution computed tomography; UIP, usual interstitial pneumonia

**Table S4. Patients with AE-RA-ILD and those with AE-IPF propensity score-matched for age, sex, baseline %FVC, P/F at AE and baseline HRCT pattern**

|  | **AE-RA-ILD**  **N = 31** | **AE-IPF**  **N = 31** | ***P*-value** |
| --- | --- | --- | --- |
| Age, years ^a^ | 72 (68–80) | 73 (67–79) | 0.6321 |
| Sex, male | 25 (80.7) | 26 (83.9) | 1.0000 |
| Smoking, ever ^a^ | 24 (77.4) | 22 (71.0) | 0.7723 |
| Baseline %FVC ^b^ | 80 (63–88) | 78 (65–88) | 0.8936 |
| Baseline %DL_CO_ ^b, c^ | 57.7 (41.0–76.1) | 66.1 (39.0–84.5) | 0.3405 |
| P/F at AE, Torr ^a^ | 254 (188–285) | 275 (200–290) | 0.4059 |
| C-reactive protein, mg/dL ^a^ | 8.3 (4.8–14.0) | 9.6 (2.5–15.4) | 0.9445 |
| KL-6, U/mL ^a^ | 1070 (820–1746) | 1415 (882–1958) | 0.5127 |
| Baseline HRCT pattern ^b^ |  |  | 0.7490 |
| UIP | 24 (77.4) | 26 (83.9) |  |
| Probable UIP | 7 (22.6) | 5 (16.1) |  |
| Indeterminate for UIP | 0 (0) | 0 (0) |  |
| Alternative | 0 (0) | 0 (0) |  |
| Treatment for first AE |  |  |  |
| Methylprednisolone pulse | 31 (100) | 31 (100) | 1.0000 |
| Prednisolone | 31 (100) | 31 (100) | 1.0000 |
| Immunosuppressant | 17 (54.8) | 14 (45.2) | 0.6119 |
| Intravenous cyclophosphamide | 11 (35.5) | 13 (41.9) | 0.7946 |
| Calcineurin inhibitor | 7 (22.6) | 1 (3.2) | 0.0529 |
| Post-AE observation period, day | 225 (27–1145) | 158 (57–711) | 0.7891 |
| Mortality within 90 days after AE onset | 13 (41.9) | 11 (35.5) | 0.7946 |
| Mortality during study period | 21 (67.7) | 26 (83.9) | 0.2351 |
| Respiratory condition-related mortality | 16 (51.6) | 22 (71.0) | 0.1919 |

Data are presented as median (interquartile range) or as number (%)

^a^ At AE onset, ^b^ within 12 months before AE, ^c^ AE-RA-ILD n = 25, AE-IPF n = 20

AE, acute exacerbation; RA, rheumatoid arthritis; ILD, interstitial lung disease; IPF, idiopathic pulmonary fibrosis; %FVC, percent predicted forced vital capacity; P/F, PaO_2_/FiO_2_ ratio; %DL_CO_, percent predicted diffusing capacity of the lung for carbon monoxide; KL-6, Krebs von den Lungen-6; HRCT, high-resolution computed tomography; UIP, usual interstitial pneumonia

**Table S5 Candidate reports**

|  | **Candidate G^2^** | **Log Worth** | **Cut-off Point** |
| --- | --- | --- | --- |
| First Split |  |  |  |
| Sex | 0.0720 | 0.1032 | Females |
| Baseline %FVC* | 14.139 | 2.6809* | 63* |
| UIP pattern on HRCT (vs. other patterns) | 1.1414 | 0.5446 | UIP |
| Age at AE onset, years | 1.8797 | 0.0652 | 71 |
| P/F at AE, Torr | 8.5574 | 1.3140 | 225 |
| Second Split |  |  |  |
| Sex | 0.7030 | 0.3960 | Females |
| Baseline %FVC | 4.6352 | 0.5403 | 79 |
| UIP pattern on HRCT (vs. other patterns) | 0.8590 | 0.4510 | UIP |
| Age at AE onset, years | 1.8625 | 0.0778 | 60.8 |
| P/F at AE, Torr* | 9.4907 | 1.5543* | 225* |

*Optimal split

Candidate G^2^, Likelihood ratio chi-square for the best split; LogWorth, the LogWorth statistics; FVC, forced vital capacity; UIP, usual interstitial pneumonia; HRCT, high-resolution computed tomography; AE, acute exacerbation; P/F, PaO_2_/FiO_2_ ratio

**Figure S1. Representative AE images in a patient with RA-ILD**

**
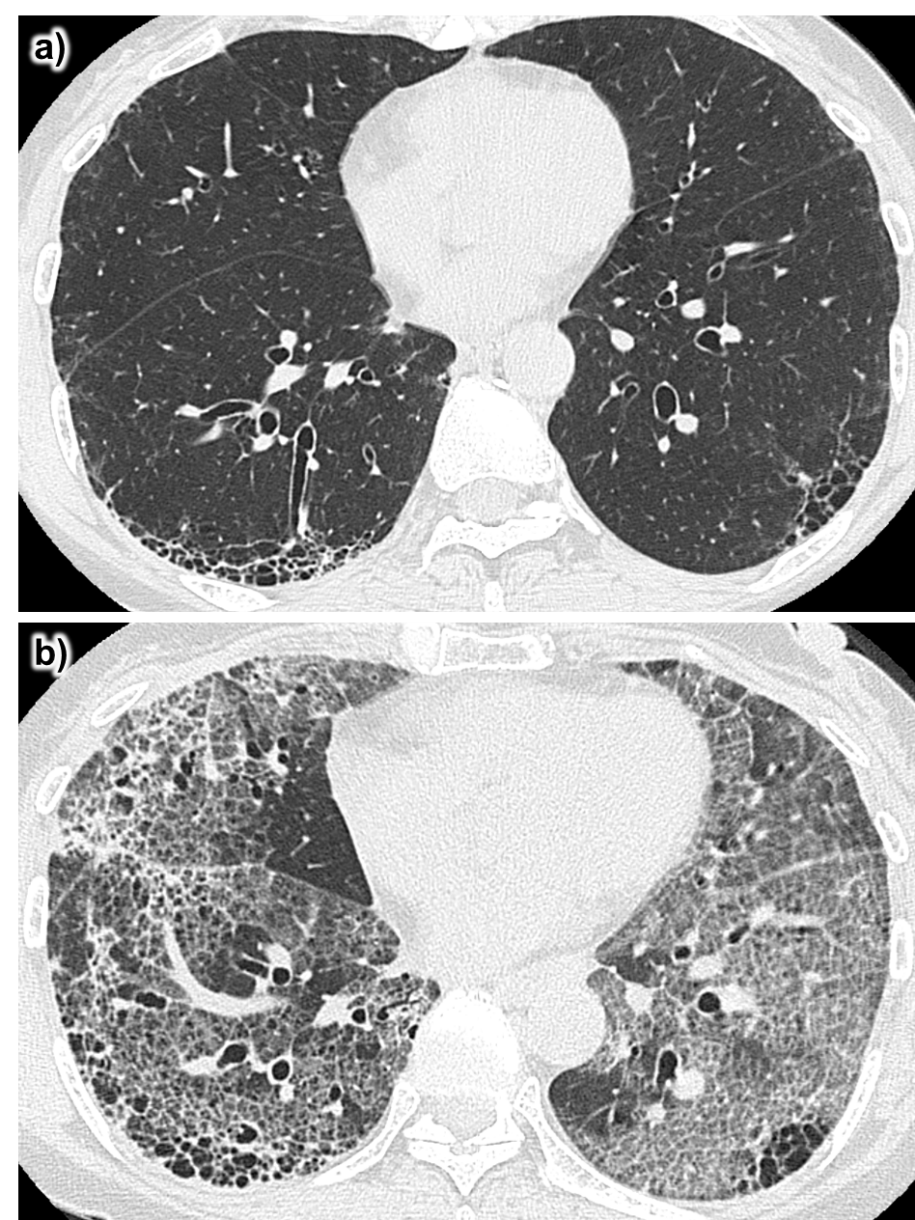
**

1. An HRCT image taken within 12 months before AE onset in a patient with RA-ILD. Reticular opacities and honeycomb, indicating fibrosing ILD, were present in the dorsal predominance of the lower lung lobes bilaterally.
2. An HRCT image at AE onset in the same patient. A new bilateral ground-glass opacity emerged superimposing on a background pattern consistent with fibrosing ILD.

AE, acute exacerbation; RA, rheumatoid arthritis; ILD, interstitial lung disease; HRCT, high-resolution computed tomography

**Figure S2. Representative image of UIP pattern on HRCT**

**
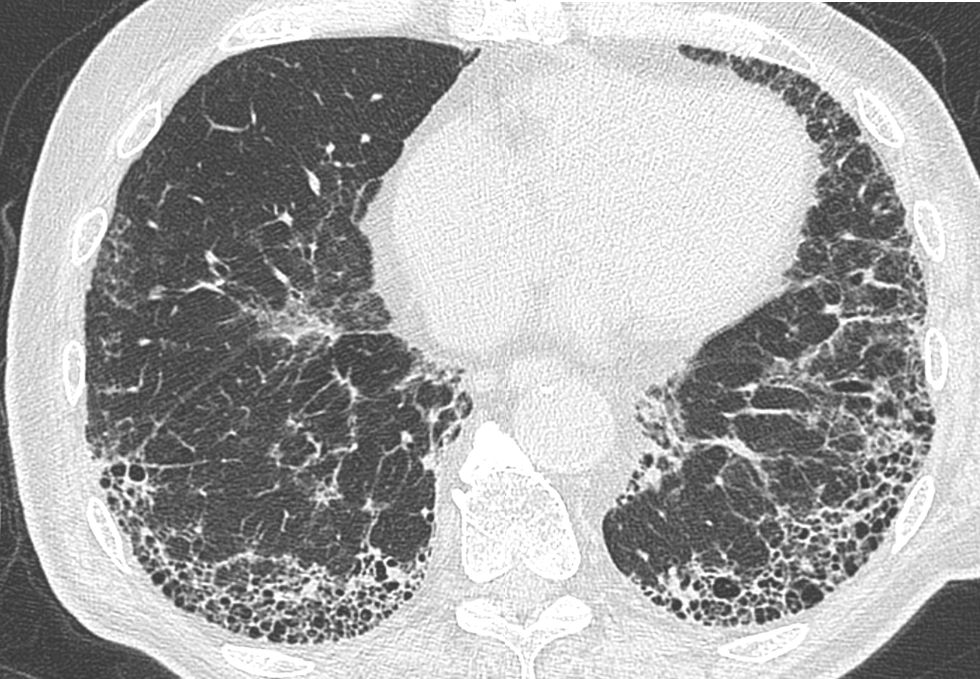
**

An HRCT image taken within 12 months before AE onset in a patient with RA-ILD.

Reticular opacities, traction bronchiectasis, and honeycombing were seen predominantly in subpleural areas bilaterally, which showed a UIP pattern on HRCT.

AE, acute exacerbation; RA, rheumatoid arthritis; ILD, interstitial lung disease; HRCT, high-resolution computed tomography; UIP, usual interstitial pneumonia
